# Evolution of the quality of life of total laryngectomy patients using electrolarynx

**DOI:** 10.1590/1806-9282.20231146

**Published:** 2024-05-03

**Authors:** Larissa Emilly Fiusa do Monte, Isabela Coelho Simão, Jodimar Ribeiro dos Reis, Plínio da Cunha Leal, Almir Vieira Dibai, Caio Márcio Barros Oliveira, Ed Carlos Rey Moura

**Affiliations:** 1 Universidade Federal do Maranhão – São Luís (MA), Brazil.

**Keywords:** Laryngeal neoplasms, Laryngectomy, Larynx artificial, Quality of life

## Abstract

**OBJECTIVE:**

Therapy and vocal rehabilitation in laryngeal cancer impact patients’ quality of life. The objective of this study was to evaluate the evolution of the quality of life of patients with laryngeal cancer submitted to total laryngectomy and using electrolarynx.

**METHODS:**

This is an observational study with a cross-sectional design and a quantitative approach. It was conducted between April 2022 and January 2023 in a Brazilian cancer hospital. For data collection, a quality of life questionnaire, validated for patients with head and neck cancer at the University of Washington, was applied in two phases: from 7 days after total laryngectomy and, subsequently, from 70 days after surgery using electronic larynx for at least 60 days. The inclusion criteria were patients undergoing total laryngectomy included on the Aldenora Bello Cancer Hospital's election list to receive the electronic larynx. Patients who did not sign the informed consent form were not included.

**RESULTS:**

The sample consisted of 31 patients, of which approximately 84% were men and approximately 93% at the age of 50 years or older. When comparing the phases, it is possible to observe that the item speech had the greatest progress, while chewing had the least. Only the item recreation, swallowing, taste, and saliva did not show any statistical significance. The score for the general quality of life questions increased.

**CONCLUSION:**

Electronic larynx is a viable and useful method of voice rehabilitation. Our data suggest that the use of the electrolarynx as a postlaryngectomy method of verbal communication is responsible for positive effects on patients’ quality of life.

## INTRODUCTION

Head and neck cancers include neoplasms of the upper aerodigestive tract, including the larynx^1^. The main risk factors are smoking and alcoholism^2^. In the larynx, most cancers develop in the glottis and supraglottic region, and their histological predominance is squamous cells^3^.

The global estimate for 2018 was 1,454,892 cases of head and neck cancers, with laryngeal cancer (LC) corresponding to 12.2%^4^. In Brazil, there is an estimate of 39,610 new diagnoses per year in the 3-year period from 2023 to 2025, with LC accounting for 20%^5^. Furthermore, the prevalence of LC is higher in men over 40 years old^3^, with low education and income and a history of smoking and drinking alcohol^1,6^.

Laryngeal cancer presents high morbidity due to the role of the larynx in voice, swallowing, and quality of life (QoL)^7^. According to the Brazilian Department of Informatics of the Unified Health System (DATASUS), diagnoses occur in advanced stages^8^. This scenario is similar to the international context and corroborates aggressive treatments associated with sequelae^9^.

Currently, conventional treatments are effective in advanced stages and include total laryngectomy (TL) with or without other modalities^7^. However, changes arising from therapy affect communication, swallowing, respiratory physiology, and psychosocial aspects^10^.

To interfere with communication and psychosocial aspects after surgery, vocal rehabilitation can be initiated with the use of devices, such as the electronic larynx (EL)^11^. This device acts by vibrating the pharyngomucosal segment, reducing patients’ anxiety due to lack of oral communication^9^.

Given the incidence of cancer and its impact on health, therapy, and vocal rehabilitation, this research study is justified by the relevance of evaluating the QoL of LC patients. In this context, the objective was to evaluate the evolution of the QoL of total laryngectomized patients using EL. Thus, the hypothesis is that there might be an improvement in those patients’ QoL.

## METHODS

### Ethical considerations

This study was approved by the Research Ethics Committee of the University Hospital of the Federal University of Maranhão in February 2022 under Opinion Number 5261052. All the participants voluntarily agreed to participate and signed the informed consent form.

### Study design

This is a quantitative cross-sectional observational study, which was held between April 2022 and January 2023 at the Aldenora Bello Cancer Hospital located in São Luís, Maranhão.

### Settings and participants

The sample consisted of 31 patients who underwent TL between April 2022 and January 2023. The inclusion criteria were patients included in the Aldenora Bello Hospital system who have the diagnosis of LC, treatment with TL, use of EL, and voluntary participation through a properly signed free and informed consent form. Patients who did not sign the informed consent form were not included. It is important to explain that the EL is the only method of vocal rehabilitation available at the mentioned hospital, which receives these devices from the local city hall.

Data collection took place in two phases: the first one from 7 days after TL and, subsequently, the second phase from 70 days after surgery using EL for at least 60 days. Patients were numbered in the order of inclusion to ensure confidentiality.

### Variables

To evaluate the progress of the intrinsic aspects of their physical and mental health, the participants were asked to answer twice, in the aforementioned interval, the QoL questionnaire, validated for patients with head and neck cancer at the University of Washington^12^. This tool addresses questions about patients’ health and QoL during the past 7 days, through 12 items: pain, appearance, activity, recreation, swallowing, chewing, speech, shoulder, taste, saliva, mood, and anxiety. Each item scores between 0 and 100; values close to the minimum indicate worse QoL, while values close to the maximum points indicate better QoL. Thus, the total score varies between 0 and 1,200. Furthermore, patients were supposed to choose at most three items considered to have the greatest impact on their life in the 7 days preceding the questionnaire. They should answer three general questions as well about QoL in the month before developing cancer, during the past 7 days, and aspects that contributed to their well-being in the last 7 days. In the end, they could also mention the problems considered to be relevant in terms of their QoL, which were not mentioned in the questionnaire.

### Statistical analysis

Ages were analyzed by mean, standard deviation, and percentage. The representation of men and women was expressed as a percentage. As for the 12 domains, the paired Student's t-test assessed the significance, by generating the p-value (p<0.05) between the mean values per question of the phases. In addition, to assess QOL, the Composite Score (the ratio between the total score of the domains and the number of domains) was calculated between the phases, being analyzed as positive the one closer to the value 100 and negative to the value 0.

The question about the most relevant problems in the past 7 days with the possibility of up to three choices and the general questions about QoL were evaluated using Fisher's exact test, which offered the p-value (p<0.05), in order to verify the significance between the phases.

## RESULTS

In the first phase, 32 participants were included. However, the final sample consisted of 31 analyses, due to the exclusion of a patient who died before the second data collection. Table 1 shows demographic details.

**Table 1:** Demographic details.

Sociodemographic characteristics (n=31)		
Age (years)	Median of age	Standard deviation
	64.6	11.6
Sex	Male	Female
	87.0%	12.9%
Occupation	Farmer	Mason
	6.4%	6.4%

n: number of patients with laryngectomies.

Table 2 shows the analysis of the domains when comparing the significance between the phases. Only the item recreation, swallowing, taste, and saliva were not statistically significant. Furthermore, Table 2 shows the most relevant issues during the past 7 days.

**Table 2:** Analysis of the 12 domains and the most relevant issues during the past 7 days.

Analysis of the 12 domains (n=31)					
First phase			Second phase		
Domains	Average score per question		Average score per question		p-value
Pain	58		87.9		**<0.001**
Appearance	65.3		85.4		**<0.001**
Activity	64.5		87.0		**<0.001**
Recreation	58		87		**<0.001**
Swallowing	63.4		77.5		0.067
Chewing	70.9		82.2		0.182
Speech	30		65.8		**<0.001**
Shoulder	79.5		94.6		**0.017**
Taste	65.6		76.3		0.182
Saliva	83.9		80.6		0.572
Humor	50.8		83.8		**<0.001**
Anxiety	52.7		93.6		**<0.001**
Most relevant issues during the past 7 days (n=31)					
First phase			Second phase		
Domains	Sample n=31	Percentage (%)	Sample n=31	Percentage (%)	p-value
Pain	8	28.8%	4	12.9%	0.335
Appearance	5	13.1%	1	3.2%	0.195
Activity	2	6.4%	2	6.4%	1
Recreation	1	3.2%	0	0%	0.990
Swallowing	8	28.8%	12	38.7%	0.415
Chewing	2	6.4%	3	9.6%	1
Speech	24	77.4%	9	29%	**<0.001**
Shoulder	3	9.6%	2	6.4%	1
Taste	1	3.2%	2	6.4%	1
Saliva	2	6.4%	7	22.5%	0.146
Humor	8	25.8%	3	9.6%	0.182
Anxiety	6	19.3%	3	9.6%	0.472

n: number of patients with laryngectomies. Statistically significant values are indicated in bold.

In Table 3, the general questions about QoL have an increase in the percentage of assertions that were evaluated as positive.

**Table 3:** General questions about quality of life.

		First phase		Second phase		
Comparison of QoL with the last month before developing cancer						
	Much better	1	3.2%	17	54.8%	**<0.001**
	Somewhat better	2	6.4%	9	29%	**0.042**
	About the same	2	6.4%	4	12.9%	0.671
	Somewhat worse	20	64.5%	0	0%	**<0.001**
	Much worse	6	19.3%	1	3.2%	0.103
QoL related to health in the last 7 days						
	Great	0	0%	2	6.4%	0.491
	Very good	0	0%	14	45.1%	**<0.001**
	Good	9	29%	13	41.9%	0.426
	Average	9	29 %	0	0%	**0.002**
	Bad	11	35.4%	2	6.4%	**0.010**
	Very bad	2	6.4%	0	0%	0.491
QoL related to factors relevant to well-being in the past 7 days						
	Great	0	0%	2	6.4%	0.419
	Very good	0	0%	12	38.7%	**<0.001**
	Good	12	38.7%	14	45.1%	0.797
	Average	10	32.2%	2	6.4%	**0.021**
	Bad	9	29.0%	1	3.2%	**0.012**
	Very bad	0	0%	0	0%	–

Statistically significant values are indicated in bold.

## DISCUSSION

The data inherent to age and sex obtained are consistent with the DATASUS information that found a higher prevalence in men and the elderly^8^. It is important to emphasize the follow-up by medical specialties jointly with other healthcare areas after TL. The speech therapy sector carried out interventions related to voice, swallowing, and breathing which are important for communication, QoL, and social and professional reintegration^13^. In addition, there is the possibility of adding other healthcare providers to the therapy, such as physical therapists, occupational therapists, and psychologists. In short, QoL is impacted by TL and requires multidisciplinary actions starting in the postoperative period^14^.

Total laryngectomy is an important resource in advanced LC, despite the physical and psychological morbidity related to respiratory and communicative changes. Furthermore, pharyngocutaneous fistula and surgical wound infection are frequent complications associated with increased length of hospital stay and the need for a new surgical intervention^15^. In this context, functional and structural changes and possible treatment complications impact patients’ QoL, mainly in the elderly^16^.

Moreover, chemotherapy and radiation therapy can be integrated into treatment and cause sequelae. The impact of radiotherapy is proportional to the number of sessions, and may cause complications, such as mucositis and tissue necrosis^17^. Therefore, the emotional and functional consequences inherent to the therapies reduce psychological and physical comfort, as chemoradiotherapy is associated with sequelae that affect the QoL^17^.

In our research study, speech presents the best comparative result between the phases. In addition to being the only item with statistical significance in the most relevant problems during the past 7 days, it is possible to infer the reduction of the negative impact of the absence of oral communication on the QoL due to the vocal rehabilitation with electrolarynx.

The possibility of adapting to aesthetic and functional changes is ratified in the evolution of appearance, activity, and recreation domains. For the latter two, it is inferred the return to a satisfactory routine without limitations in carrying out activities relevant to daily life and well-being.

Mood and anxiety, after treatment and process of adapting to changes, improve and are relevant given the impact on mental health justified by the association between vocal changes and poor communication that contribute to social isolation^17^. Therefore, EL enables dialogue in the absence of oral communication.

In line with the evolution of the activity item, a Spanish study found that most patients remained active after TL, with a relationship with vocal rehabilitation. To the new vocal condition, the emotional aspects advance together with the social function^18^. In this study, the statistical approval of the item's mood, anxiety, activity, and recreation is in agreement. The progress of the issue on QoL related to activities inherent to well-being is considered a means of admitting the relevance of vocal rehabilitation.

In a research study by the National Cancer Institute with patients undergoing treatment for LC, despite different stages and therapies, responses in the domains of swallowing, chewing, taste, and saliva were not considered significant^9^, as well as in our study. It is important to mention the early intervention in adapting to changes inherent to swallowing and chewing.

Another study at the University of North Carolina found that survivors of head and neck squamous cell carcinoma are affected by mental disorders associated with greater pain and negative QoL results^19^. Anxiety, in addition to general questions about QoL, is concordant, since in the first phase, which presents a short interval with TL, these questions are affected concomitantly.

In patients with head and neck cancers, pain is common after curative treatment. Among the most affected areas, the shoulder is mentioned, and arm disability may coexist. Widespread distribution of pain is frequent and there may not be limitations to areas irradiated in radiotherapy treatments. However, multimodal interventions and pain treatment reduce awareness^20^. It is possible to relate to the retrogression in the intensity and presence of pain and alterations in the shoulder between the phases.

In the evaluation of the composite score, there is an improvement between the phases, and this scenario is confirmed by the evolution of positive answers in the general questions and in the pain, mood, and anxiety domains. The progress with longer intervals after surgery and rehabilitation is confirmed.

Laryngeal cancer and treatment change how patients see themselves, interact, and play their social role^19^. Despite the favorable results, adjuvant treatments and relapses have an impact on the evolution. In this sample, in addition to the exclusion of the deceased patient, there was the death of four individuals who completed the two phases. On the contrary, it is important to consider the improvement after discharge from clinical treatment.

Despite the methodological limitations inherent to the design, the results were able to describe important aspects of the QoL of this population.

## CONCLUSION

Electrolarynx is a viable and useful method of voice rehabilitation for patients who have had laryngectomies, and according to these data, it is responsible for positive effects on patients’ QoL. Significant results were observed for the pain, appearance, activity, recreation, speech, shoulder, mood, and anxiety domains. In addition, general questions about QoL progressed. However, the swallowing, chewing, taste, and saliva domains were rejected in the statistics. Furthermore, only speech showed statistical significance in the comparison between the phases of the most relevant problems during the past 7 days.
